# Reliability and accuracy of dental MRI for measuring root canal length of incisors and canines: a clinical pilot study

**DOI:** 10.1038/s41598-022-17889-3

**Published:** 2022-08-18

**Authors:** Mousa Zidan, Franz S. Schwindling, Alexander Juerchott, Johannes Mente, Mathias Nittka, Zahra Hosseini, Sabine Heiland, Martin Bendszus, Tim Hilgenfeld

**Affiliations:** 1 Department of Neuroradiology, Heidelberg University Hospital, Im Neuenheimer Feld 400, 69120 Heidelberg, Germany; 2 Department of Prosthodontics, Heidelberg University Hospital, Heidelberg, Germany; 3 Division of Endodontics and Dental Traumatology, Department of Conservative Dentistry, Heidelberg University Hospital, Heidelberg, Germany; 4 Magnetic Resonance, Siemens Healthcare GmbH, Erlangen, Germany; 5 Magnetic Resonance R&D Collaborations, Siemens Medical Solutions, Atlanta, GA USA; 6 Division of Experimental Radiology, Department of Neuroradiology, Heidelberg University Hospital, Heidelberg, Germany

**Keywords:** Cone-beam computed tomography, Dental radiology, Dentistry, Endodontics, Root canal treatment, Magnetic resonance imaging, Three-dimensional imaging, Medical imaging, Computed tomography

## Abstract

To evaluate whether high-resolution, non-contrast-enhanced dental MRI (dMRI) can reliably and accurately measure the canal length of incisors and canines compared with cone-beam computed tomography (CBCT). Three-Tesla dMRI was performed in 31 participants (mean age: 50.1 ± 14.2 years) with CBCT data. In total, 67 teeth were included (28 from the upper jaw and 39 from the lower jaw; 25 central incisors, 22 lateral incisors, and 20 canines). CBCT and dMRI datasets were reconstructed to visualize the root canal pathway in a single slice in the vestibulo-oral (V-O) and mesio-distal (M-D) direction. Root canal length was measured twice by two radiologists using dMRI and CBCT. Data were statistically analyzed by calculating intraclass correlation coefficients (ICCs) and performing Bland–Altman analysis. The reliability of dMRI measurements was excellent and comparable to that of CBCT measurements (intra-rater I/intra-rater II/inter-rater was 0.990/0.965/0.951 for dMRI vs. 0.990/0.994/0.992 for CBCT in the M-D direction and 0.991/0.956/0.967 for dMRI vs. 0.998/0.994/0.996 for CBCT in the V-O direction). According to Bland–Altman analysis, the mean (95% confidence interval) underestimation of root canal lengths was 0.67 mm (− 1.22 to 2.57) for dMRI and 0.87 mm (− 0.29 to 2.04) for CBCT in the M-D direction/V-O direction. In 92.5% of cases, dMRI measurements of canal length had an accuracy within 0–2 mm. Visualization and measurement of canal length in vivo using dMRI is feasible. The reliability of dMRI measurements was high and comparable to that of CBCT measurements. However, the spatial and temporal resolution of dMRI is lower than that of CBCT, which means dMRI measurements are less accurate than CBCT measurements. This means dMRI is currently unsuitable for measuring canal length in clinical practice.

## Introduction

Since the introduction of cone-beam computed tomography (CBCT) in the late 1990s^1^ three-dimensional (3D) imaging has shown great potential for dentalmaxillo-facial imaging^2^. CBCT can accurately depict endodontic tooth anatomy, the number of root canals, and details of the surrounding alveolar bone, allowing it to detect pathologies that would otherwise be missed in periapical intraoral radiographs^3,4^. Therefore, CBCT has become an essential tool in endodontics for diagnosis, treatment planning, and evaluating treatment outcomes^5,6^.

Accurate filling of root canals determines the success of root canal treatment^7,8^ several studies have evaluated whether CBCT can be used to determine the canal length and whether CBCT measurements are more precise than electronic apex locator (EAL) measurements^9–12^. These studies concluded that CBCT imaging is accurate enough to determine the canal length.

However, EAL remains the gold standard to determine accurate length measurements in most cases. But there are also a few exceptions in this regard. Limitations for EAL measurements are given in cases like metallic restorations^13^, partially or totally obliterated root canals^14^, root perforations^15^ or measurements during root canal retreatments^16^. The aim should always be to combine EAL measurements with imaging techniques of proven accuracy^17^.

Despite the benefits of CBCT over intraoral periapical radiographs, its application is limited by the high radiation dose^18,19^. A radiation-free imaging modality is needed, especially for young patients who are most radiosensitive^20^. Dental magnetic resonance imaging (dMRI) has excellent soft-tissue contrast and is a promising imaging modality for diagnosing various pathologies in endodontics^21–24^, including promising results in differentiating periapical lesions^25–27^, orthodontics^28^, craniomaxillofacial surgery^29^, and implantology^30–34^.

Despite promising ex vivo results^35–37^, the ability of dMRI to measure root canal length has not been evaluated in vivo. The development of dedicated coil and sequence techniques^38–41^ has increased the resolution of dMRI and has reduced the number of artifacts observed during clinical imaging. Thus, we hypothesized that dMRI may be a reliable and accurate alternative to CBCT for measuring root canal length. To test this hypothesis, we performed a prospective clinical study to compare dMRI measurements with corresponding CBCT measurements.

## Materials and methods

This prospective study was approved by the ethics committee of the University of Heidelberg (approval number S-404/2014) and written informed consent was obtained from all participants. Inclusion criteria were the presence of incisors and canines and a CBCT scan, which had been performed for clinical reasons such as implant planning. Patient-related exclusion criteria were < 18 years of age, implants not safe for 3 T MRI, pregnancy, and claustrophobia. Tooth-related exclusion criteria were dMRI/CBCT artifacts that compromised pulp, poor visibility of the root canal or incisal edge, previous root canal treatment, and crowns. The sample size was calculated according to recommendations for clinical pilot studies^42^ and patients were consecutively recruited in a single center. Throughout the study dMRI measurements were directly compared to corresponding CBCT measurements.

### Ethical approval and consent

The use of human participants in this study was approved by the ethics committee of the University of Heidelberg and the study was performed in accordance with the 1964 Declaration of Helsinki and its later amendments. All patients gave written informed consent prior to inclusion in this study.

### Imaging

All CBCT images were acquired using a 3D Accuitomo 170 scanner (J Morita; Kyoto, Japan) with the following acquisition parameters: field of view: 8 × 8 cm^2^, tube voltage: 90 kV, tube current: 7 mA, 14-bit, 360° rotation in 17 s, 560 frames, and an isotropic voxel size of 160 μm. All MRI scans were performed on a 3 T MRI system (Magnetom Trio; Siemens Healthineers GmbH, Erlangen, Germany) using a dedicated 15-channel dental surface coil (Mandibula, Noras MRI products GmbH). A single PD-weighted 3D MSVAT-SPACE (multiple slab acquisition with view angle tilting gradient based on a sampling perfection with application-optimized contrasts using different flip angle evolution) sequence was applied. This sequence was optimized for dental MRI as previously described by Hilgenfeld et al.^39^. Sequence parameters were 6.4 ms echo time; 1170 ms repetition time; 168 mm × 131 mm field of view; 384 × 300 acquisition matrix; 0.44 mm × 0.44 mm × 0.44 mm voxel size; 220% slice oversampling; 80 slices; and 7:45 min acquisition.

### Image analysis

**Figure 1:**
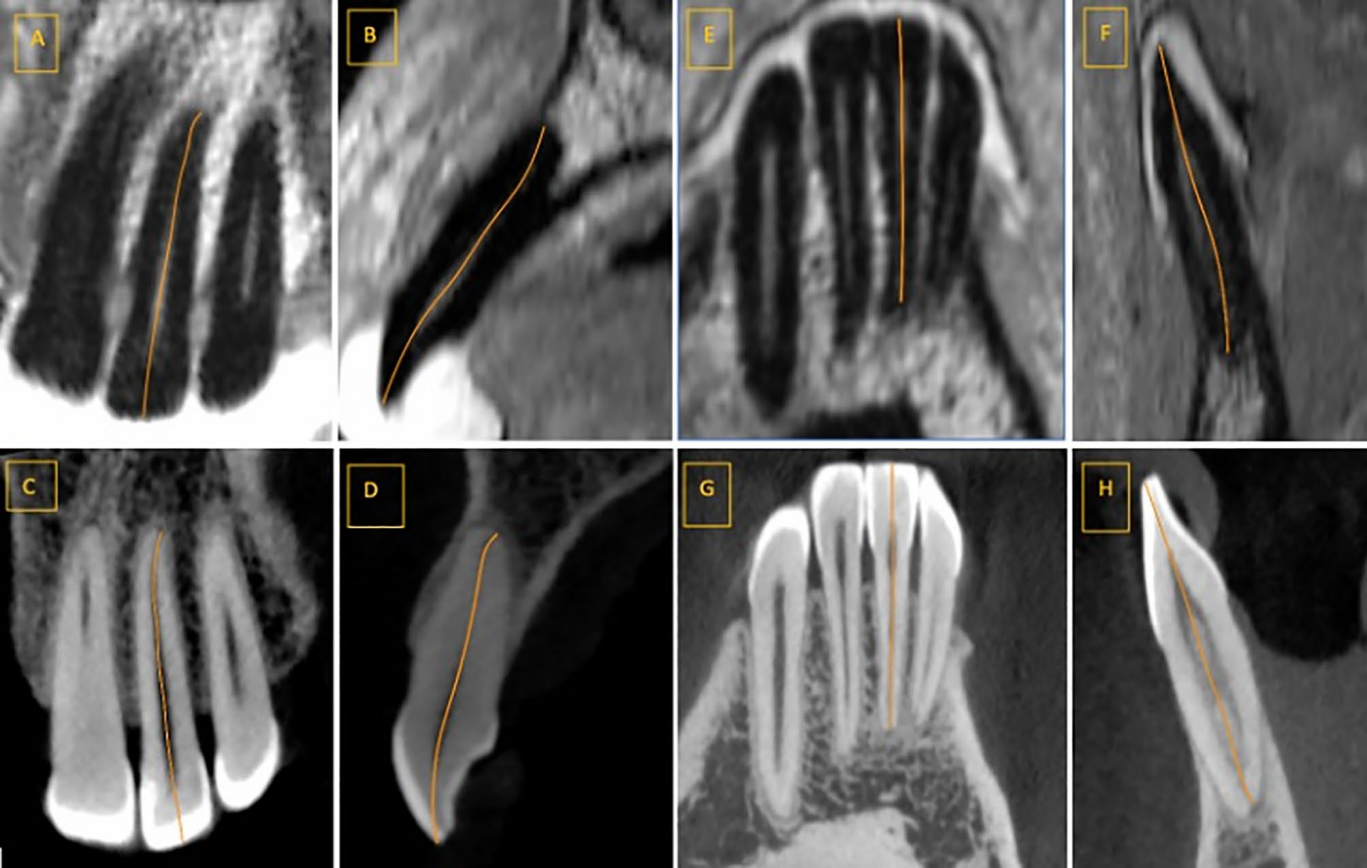
Canal length measurements by dMRI (upper row) and CBCT (lower row) in the mesio-distal (M-D) and vestibulo-oral (V-O) directions in the second left upper incisor (**A–D**) and the first right lower incisor (**E–H**). dMRI slices of a second incisor in the left upper jaw in the M-D direction (**A**) and the V-O direction (**B**). (**C,D**) Are the corresponding CBCT slices. dMRI slices of a first incisor in the right lower jaw in the M-D direction (**E**) and the V-O direction (**F**). (**G,H**) Are the corresponding CBCT slices.

CBCT and MRI datasets were reconstructed using Osirix (v. 8.5.1., Geneva, Switzerland) to visualize the root canal pathway in a single slice in the vestibulo-oral (V-O) and mesio-distal (M-D) direction as previously described^11^. Next, images were randomized and evaluated twice by two experienced radiologists with an interval of 2 weeks between evaluations to exclude learning bias. CBCT and MRI datasets were also analyzed separately in each round, with an interval of 2 weeks between the imaging modalities. CBCT and MRI datasets were analyzed in different random orders. The contrast/saturation of the images could be adjusted. The root canal length was defined as the distance between the most incisal edge in the projected midline of the pulp cavity and the major foramen (Fig. 1). Curved measurement lines were placed in the pulpal center, including curvatures of the canal as previously described^11^.

### Statistical analysis

Statistical analysis was performed using SPSS 23 (SPSS Inc., Chicago, USA). To assess the reliability of CBCT and MRI measurements, intra- and inter-rater agreement were calculated using intraclass correlation coefficients (ICCs) with 95% confidence intervals (CIs). The accuracy of MRI measurements was determined using Bland–Altman plots, illustrating differences between corresponding dMRI and CBCT values. We also calculated the average difference in measurements between dMRI and CBCT and the upper and lower limit of the 95% CI for the two modalities. To allow comparison of our data with those of previous studies, we also calculated the Pearson correlation coefficient between dMRI and CBCT measurements.

## Results

In total, 31 participants (13 males, 18 females) of white European ethnicity were enrolled in the study. The mean ± standard deviation (SD) age was 50.1 ± 14.2 years (median 51.0; range 28–74). Teeth with movement artifacts or metal artifacts were excluded, leaving 67 teeth (28 in the upper jaw, 39 in the lower jaw; 25 first incisors, 22 s incisors, 20 canines) in the final assessment. High-resolution 3D dMRI produced detailed images of pulp and root canals (Fig. 1).

### Reliability

Intra- and inter-rater reliability of root canal length measurements were excellent for both modalities (range 0.951–0.998) (Tables 1, 2). CBCT measurements had a slightly higher intra-rater agreement than dMRI measurements did (range CBCT: 0.990–0.998; range dMRI: 0.956–0.991). A similar trend was observed for the inter-rater agreement, with higher values for CBCT (M-D: 0.992, 95% CI: 0.986–0.996; V-O: 0.996, 95% CI: 0.994–0.998) than for dMRI (M-D: 0.951, 95% CI: 0.921–0.970; V-O: 0.967, 95% CI: 0.943–0.981).

**Table 1:** Intra-rater ICC and 95% confidence interval of CBCT and dMRI canal length measurements in the mesio-distal (M-D) and vestibulo-oral (V-O) directions.

	Rater I				Rater II			
	CBCT		dMRI		CBCT		dMRI	
	M-D	V-O	M-D	V-O	M-D	V-O	M-D	V-O
Intra-rater	**0.990** (0.984–0.994)	**0.998** (0.997–0.999)	**0.990** (0.984–0.994)	**0.991** (0.985–0.995)	**0.994** (0.990–0.996)	**0.994** (0.989–0.997)	**0.965** (0.943–0.978)	**0.956** (0.928–0.973)

Significant values are in bold.

**Table 2:** Inter-rater ICC and 95% confidence interval of CBCT and dMRI canal length measurements in the mesio-distal (M-D) and vestibulo-oral (V-O) directions.

	CBCT		dMRI	
	M-D	V-O	M-D	V-O
Inter-rater	**0.992** (0.986–0.996)	**0.996** (0.994–0.998)	**0.951** (0.921–0.970)	**0.967** (0.943–0.981)

Significant values are in bold.

### Accuracy

**Figure 2:**
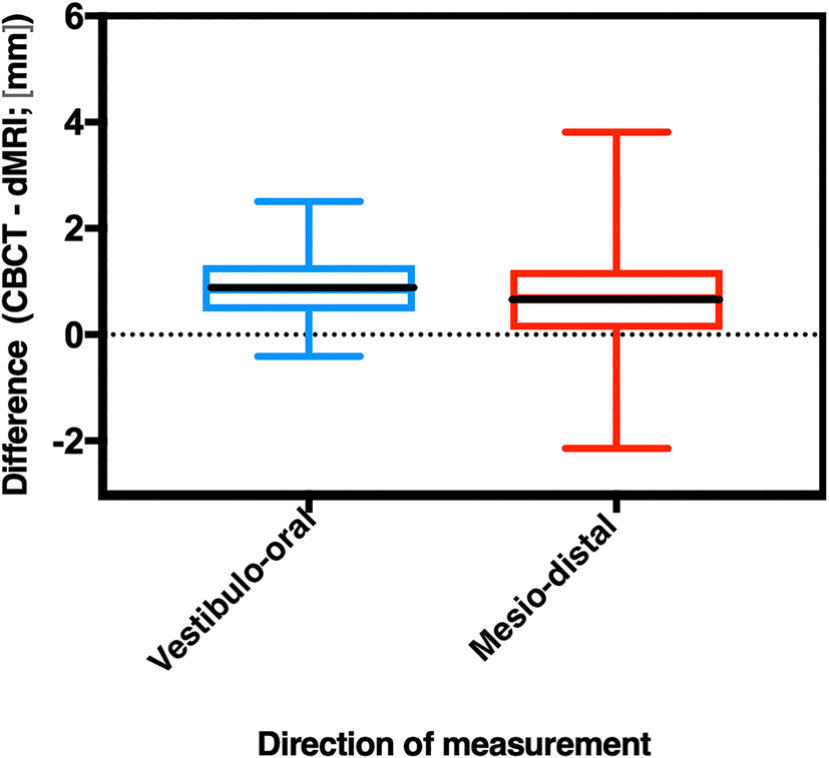
Box plots with 95% CIs of differences between dMRI and CBCT canal length measurements. The bold black line represents the median differences.

dMRI measurements underestimated the canal length compared to the CBCT measurements (underestimation in 95.5% of dMRI measurements in the V-O direction and in 79.1% of dMRI measurements in the M-D direction). The median ± SD underestimation was 0.84 ± 0.59 mm in the V-O direction and 0.69 ± 0.96 mm in the M-D direction (Fig. 2). The proportion of dMRI measurements that were 0–2 mm different from CBCT measurements was 92.5% in the V-O direction and 71.6% in the M-D direction. The canal length was overestimated beyond 0.5 mm of the apex in 10.4% of dMRI measurements in the M-D direction compared to the CBCT measurements. The maximum difference between dMRI measurements and the corresponding CBCT measurements was 2.51 mm underestimation and 0.41 mm overestimation in the V-O plane and 3.81 mm underestimation and 2.14 mm overestimation in the M-D plane.

**Figure 3:**
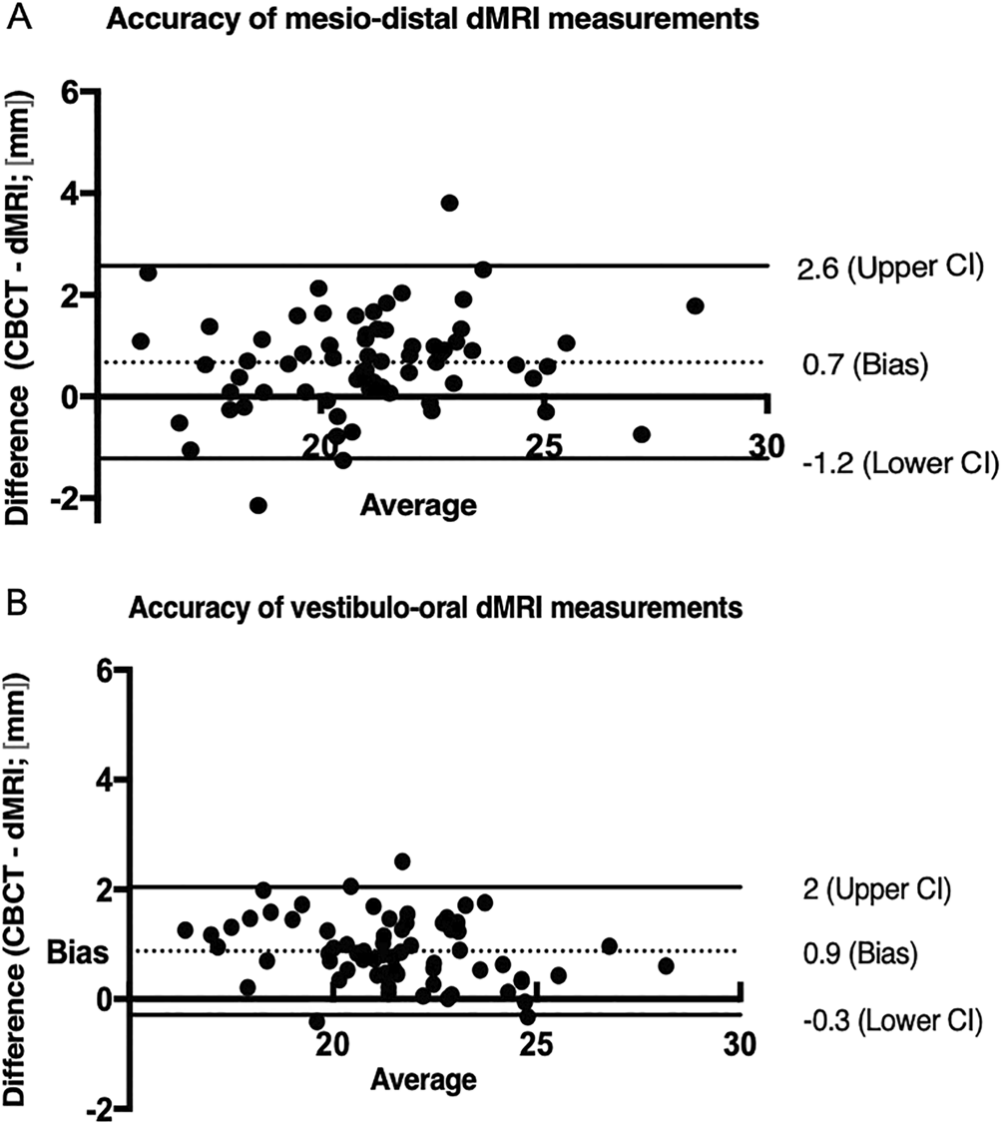
Bland–Altman plots, illustrating differences between corresponding CBCT and dMRI canal length measurements in the mesio-distal (**A**) and vestibulo-oral (**B**) plane. The dotted line represents the mean differences (bias) and the solid lines represent the upper and lower 95% limits of agreement.

Bland–Altman analysis revealed consistent underestimation of canal lengths by dMRI compared to the CBCT measurements. The mean underestimation (lower/upper limits of agreement) of root canal length was 0.67 (− 1.22 to 2.57)/0.87 (− 0.29 to 2.04) mm for M-D and V-O measurements (Fig. 3).

The accuracy of dMRI measurements was similar between the upper and lower jaw. Bland–Altman analysis revealed an underestimation (95% limits of agreement) of 0.67 (− 0.88 to 2.24)/0.74 (− 0.47 to 1.97) mm for the upper jaw and 0.67 (− 1.45 to 2.80)/0.96 (− 0.13 to 2.06) mm for the lower jaw in the M-D and V-O directions.

## Discussion

In this study, we compared the reliability and accuracy of root canal working lengths measured by dMRI and CBCT in vivo. The reliability of dMRI canal length measurements was similar to that of CBCT measurements in both studied directions. However, dMRI canal length measurements were less accurate than CBCT measurements in both directions 0.67 mm (− 1.22 to 2.57) for mesio-distal measurements and 0.87 mm (− 0.29 to 2.04) for vestibulo-oral measurements. These results are clinically important because they reflect how CBCT-based 3D imaging has dramatically improved diagnostic accuracy and treatment planning in endodontics^5,43^. However, CBCT uses higher radiation doses than conventional radiography, so is limited. A comparable radiation-free 3D imaging modality like dMRI would be of great value.

A methodological strength of this study is the evaluation of dMRI reliability and accuracy in vivo. This approach incorporated clinically relevant factors like movement and metal artifacts and tooth variations, which play a crucial role in the outcome of endodontic therapy^8,44^. Most previous evaluations of CBCT measurements have been performed ex vivo^9,12^. Liang et al. and Connert et al. included 162 and 42 extracted teeth, respectively. Other studies that were performed in vivo have been limited by their sample size^10,11^: Janner et al. and Jeger et al. included 9 and 40 teeth, respectively. Our in vivo study included a larger sample size than that of previous studies (67 teeth, 134 tooth datasets, and 1072 root canal measurements) and we analyzed teeth in the upper and lower jaw separately, which was not possible in previous in vivo studies because of the low sample size. This is relevant because pulp sizes differ between the upper and lower jaw, and this difference may affect the accuracy of dMRI measurements.

The intra- and inter-reliability of dMRI measurements were excellent and comparable to those of CBCT measurements. Reliability of CBCT measurements in previous studies was measured as an ICC of 0.982 under ex vivo conditions^10^ and a Pearson correlation coefficient of 0.99 under in vivo conditions^10^. In agreement, we also observed high ICCs for CBCT measurements (range 0.990–0.998) as well as for dMRI measurements (range 0.956–0.991). These results show that the reliability of dMRI measurements is comparable to that of CBCT measurements.

We investigated the root canal length in M-D and V-O directions and found a lower mean error in the M-D direction (mean difference 0.67 mm) than in the V-O direction (mean difference 0.87 mm). This result is in accordance with the results of a previous in vivo CBCT study by Jeger et al., who found a slightly lower mean error in the M-D direction than in the V-O direction (0.48 mm vs. 0.49 mm)^10^.

Previous studies have shown a high accuracy of CBCT root canal length measurements compared with electronic apex locator measurements. Pearson correlation coefficients for CBCT measurements compared with electronic apex measurements were 0.977 in ex vivo studies^9^ and 0.968–0.97 for in vivo studies^10,11^. We found a similarly high correlation between dMRI and CBCT measurements (0.927–0.969) in the present study.

We also investigated measurement error in canal length measurements. In previous studies of CBCT canal length measurements, Janner et al. reported errors of 0.4 mm (range 0.03–1.6 mm) and Jeger et al. reported errors of 0.51 mm (0.02–1.83 mm) under clinical conditions^10,11^. In agreement, Connert et al. observed an error of 0.41 mm (0.31–0.52 mm) and Liang et al. an error of 0.46 mm (0.41–0.50) under ex vivo conditions^9,12^. Our dMRI measurements showed a lower accuracy and a larger range of error of 0.67 mm (− 1.22 to 2.57) than these earlier CBCT measurements did. Although up to 92.5% of our measurements were within an error range of − 2 mm to 0 mm, there were some outliers. This shows that dMRI is currently not accurate enough to measure the canal length because instrumentation and root filling should be within 0–2 mm of the radiographic apex^7^. However, dMRI measurements were still within the magnitude of CBCT measurements, despite the higher spatial resolution (factor of voxel volume of 21) and longer acquisition time (factor 26) of CBCT.

Several limitations must be acknowledged in this study. First, we chose CBCT measurements and not electronic apex locator measurements as the reference modality. However, previous studies observed a high accuracy of CBCT-based measurements compared with clinical gold standard EAL. Second, although we chose a high-resolution dMRI setup, the spatial resolution as well as acquisition time of dMRI were substantially lower than that of CBCT, resulting in lower accuracy. Third, metallic artifacts due to dental restorations as well as claustrophobia due to narrow coils are relevant limitations in dMRI. These limitations, however, might be reduced with optimized MRI sequence techniques for metal artifact reduction^39^ and intraoral placement of MRI coils in the future^45^. Finally, the costs and availability of MRI machines also limit the clinical application of dMRI and these obstacles should be addressed in future research.

## Conclusion

This prospective in vivo study has demonstrated that measuring root canal length in incisors and canines in vivo using dMRI is feasible. The reliability of dMRI canal length measurements was comparable to that of CBCT measurements. However, dMRI measurements were less accurate because of lower resolution and longer acquisition times, which currently hampers its clinical application. Further research is needed to increase the spatial resolution and reduce the acquisition time of dMRI before this promising non-ionizing imaging modality can be used to measure canal length under clinical conditions.

## Data Availability

The datasets generated during and/or analyzed during the current study are not publicly available because of data privacy protection of patients and participants but are available from the corresponding author on reasonable request.
